# Growth and body composition of adolescents and young adults with perinatal HIV infection: a systematic review and meta-analysis

**DOI:** 10.1186/s12889-025-21838-w

**Published:** 2025-02-21

**Authors:** Priscila R. F. Costa, Nathalia S. Guimarães, Carlos R. N. Lira, Luana O. Leite, Rita de Cássia Ribeiro da Silva, Maurício L. Barreto, Enny S. Paixão

**Affiliations:** 1 Department of Science Nutrition, Federal University of Bahia, 32 Araujo Pinho Avenue, Salvador, 40.110-907 Brazil; 2 Post-Graduation Program in Food, Nutrition and Health, Federal University of Bahia, Salvador, Brazil; 3 Center for Data and Knowledge Integration for Health (CIDACS), Oswaldo Cruz Foundation, Salvador, Brazil; 4 Department of Nutrition, Federal University of Minas Gerais, Belo Horizonte, Minas Gerais, Brazil; 5 Department of Epidemiology and Population Health, London School of Hygiene and Tropical Medicine, London, UK

**Keywords:** Systematic review, Adolescent growth, HIV, Body composition, Perinatal HIV infection

## Abstract

**Introduction:**

Approximately 1.3 million pregnancies are exposed to HIV perinatally each year, with transmission rates ranging from 4 to 21%, despite 85% antiretroviral therapy (ART) coverage among pregnant women. While ART has significantly reduced mother-to-child transmission, recent studies indicate an increased risk of chronic conditions among perinatally HIV-infected youth. There is a critical need for further research on the growth and health of these populations to inform effective health interventions. Therefore, the aim of this study is to critically evaluate observational research assessing the growth and body composition of perinatally HIV-infected adolescents and young adults.

**Methods:**

We conducted a systematic review using the MEDLINE (by PubMed), Embase, Central (by Cochrane Library), Latin American and Caribbean Health Science Information (LILACS) and Web of Science databases. The initial search was conducted on November 24, 2022, and updated on June 3, 2024. We included cohort studies that evaluated perinatally HIV-infected adolescents (aged 10 to 19 years) and young adults (aged 20 to 24 years). A meta-analysis was performed to estimate the prevalence of stunting (HAZ < -2) and underweight (BAZ < -2). Additionally, we conducted meta-analyses for the mean values of HAZ and BAZ in the population after the follow-up period, as well as for the mean change in HAZ and BAZ post-follow-up.

**Results:**

Our search strategy retrieved 11,017 documents. After excluding duplicates, we analyzed 9,273 titles and abstracts. A full-text review of the remaining 87 records was conducted. Following the updated search, we identified 6 publications from 4 studies, resulting in a total of 14 studies and 16 publications. Nine studies focused exclusively on adolescents, while three evaluated both children and adolescents, and three included both adolescents and young adults. We estimated a prevalence of stunting at 26% (95% CI: 0.23–0.29) and underweight at 14% (95% CI: 0.12–0.17). In this population, the mean HAZ was -1.58 (95% CI: -1.90; -1.27), and the mean BAZ was -0.34 (95% CI: -0.61; -0.06). Additionally, we identified an increase of 0.55 (95% CI: 0.07; 1.03) in mean HAZ and 0.12 (95% CI: -0.56; 0.79) in mean BAZ after the follow-up period.

**Conclusions:**

Our study highlights significant growth and body composition challenges among perinatally HIV-infected adolescents and young adults, with concerning rates of stunting and underweight. Although there was a modest increase in height-for-age, persistent challenges in achieving optimal growth remain. The slight improvement in BMI is insufficient to fully address underweight concerns. The limited number of studies and their inherent limitations restrict the ability to draw consistent conclusions regarding the effects and magnitude of exposure on anthropometric outcomes. Further research is needed to better elucidate these relationships.

**Trial registration:**

CRD42022372837.

**Supplementary Information:**

The online version contains supplementary material available at 10.1186/s12889-025-21838-w.

## Introduction

According to the World Health Organization, approximately 1.3 million pregnancies are exposed to HIV perinatally each year worldwide [1]. While highly effective interventions have significantly reduced mother-to-child transmission rates—potentially as high as 45% without intervention—vertical transmission continues to occur. Despite antiretroviral therapy (ART) coverage reaching an estimated 85% among pregnant women living with HIV [1], transmission rates still range between 4 and 21% in priority countries [2].

The use of ART during pregnancy has been pivotal in lowering transmission rates, resulting in the birth of an estimated 15.4 million HIV-exposed but uninfected (HEU) children globally each year [3–5]. However, the long-term effects of universal access to ART and prolonged breastfeeding on these children are not well understood. Research suggests that ART exposure may contribute to adverse birth outcomes, such as preterm birth and low birth weight [6]. Furthermore, HEU children have been reported to experience higher rates of mortality, morbidity, stunting, and developmental delays compared to their HIV-unexposed peers [7].

A longitudinal study demonstrated differences in mitochondrial-related measures and insulin resistance were observed in youth living with perinatally-acquired HIV vs youth perinatally HIV-exposed but uninfected [8]. Haw and collaborators (2024) [9] identified a high incidence of diabetes mellitus type 2 (T2DM), hypercholesterolemia, hypertriglyceridemia, hypertension, and chronic kidney disease (CKD) among youth living with perinatally acquired HIV, highlighting that earlier screening at younger ages may be an important strategy to strengthen prevention measures and initiate timely treatment.

A perinatally HIV-infected child now faces a chronic disease rather than a progressive, fatal one, allowing many to reach adolescence and young adult. A systematic review also found that these children tend to have lower weight and length Z-scores at birth and face elevated risks of morbidity and mortality from infectious diseases [10]. Systematic review and meta-analyses [11] presented growth data for HIV-exposed but uninfected children, but those for infected children are not established for decision-making. Given the limited and sometimes conflicting data on the growth and body composition of PHIV adolescents and young exposed perinatally HIV-infected this study seeks to critically evaluate observational research on this topic.

## Methods

This systematic review and meta-analysis was based on recommendations from the Cochrane Guidelines for Systematic Reviews of Interventions [12] and was written according to the Preferred Reporting Items for Systematic Reviews and Meta-Analyses (PRISMA) guidelines [13]. The review protocol was registered at PROSPERO (CRD42022372837).

### Search strategy

To identify observational studies evaluating the effects of perinatally HIV infection on the growth and body composition of adolescents (from 10 to 19 years old) and young adults (from 20 to 24 years old), we searched five independent databases to perform a sensitive literature search: MEDLINE (by PubMed), Embase, Central (by Cochrane Library), Latin American and Caribbean Health Science Information (LILACS) and Web of Science.

There was no language, date, document type, publication status or geographic restriction for inclusion of records. The search was conducted in 24th November 2022 and updated in 3th June 2024. Descriptors were identified in Medical Subject Headings (MeSH), Descritores em Ciências da Saúde (Decs) and Embase Subject Headings (Emtree). We used the following terms to search: “HIV”; “HIV Infections”; “HIV-1”; “HIV-2”; “Anti-Retroviral Agents”; “Infants”; “Child”; “Adolescents”; “Young Adult”; “Growth and Development”; “Growth”; “Body Size”; “Body composition”; “Body Fat Disturbance”; “Obesity”; “Overweight”. The search strategy was adapted based on descriptors in each database and is presented in the Supplementary material [Supporting Information].

### Outcomes

The primary outcome was growth evaluated by height-for-age (HAZ), weight-for-height/length (WHZ) and weight-for-age (WAZ). The secondary outcome was the occurrence of underweight and obesity evaluated by BMI-for-age (BAZ) or BMI, % body fat, % lean mass, and waist circumference.

### Eligibility criteria

We included cohort studies that evaluated adolescents and young adults who were perinatally HIV-infected. Reviews, in vitro studies, experimental studies, interventional studies (testing new drugs), and editorials were excluded.

### Study selection and data extraction

Electronic search results from defined databases were uploaded to the Rayyan Qatar Computing Research Institute. Study selection and data extraction were independently performed by two investigators. A third reviewer resolved any disagreements. Authors initially screened titles and abstracts. Subsequently, they assessed each study to determine whether it met the inclusion criteria.

We extracted data on study design (methods, location, setting, inclusion/exclusion criteria, duration and number of participants in each group), participant characteristics (sample size, common population demographics variables, treatment and characteristics from the beginning of the study), outcome information (association measures, mean, standard deviation, median, interquartile interval, *p* value, confidence interval), methods of outcome data collection (weight, height and waist circumference methods), statistical analysis, study limitations, and main conclusions.

### Quality assessment

Two investigators independently assessed the risk of bias in the selected studies according to the Joanna Briggs Institute Critical Appraisal checklist for cohort studies. The included studies were classified as having “high risk of bias” when the study's “yes” score was between 0 and 49%; “moderate risk of bias” when the study was scored “yes” between 50 and 69%; and “low risk of bias” when the study 'yes' score was ≥ 70% [14]. Judging was also performed by two reviewers independently, and disagreements were resolved by a third reviewer. The risk of bias results were presented descriptively and tabulated (Table 2).

### Statistical analysis

For the data synthesis, we adopted a narrative review for the qualitative data. For the combinable studies, we undertook three different types of meta-analyses. One used the random effects model conducted with the Stata metaprop command to estimate the prevalence of stunting (HAZ < −2) and underweight (BAZ < −2) in individuals perinatally HIV-infected. It allows you to compute 95% confidence intervals using the statistical score and the exact binomial method and incorporates the Freeman-Tukey arcsine double proportions transformation. This method also allows us to model the intra study variability using the binomial distribution. That is, it makes the data distribution normal and stabilizes the variances [15]. The inverse function of the double arcsine transformation was also derived in the literature to recover the original proportion scale after data aggregation [15], maintaining the interpretability of the final result. Thus, the summary prevalence of stunting and underweight was generated, as well as its respective 95% confidence interval.

Meta-analyses for the global mean of HAZ and BAZ in the population after the follow-up period and for the mean change of HAZ and BAZ after the follow-up (by subtracting the final mean from the initial mean) were calculated converting the median, first and third quartiles, and the number of subjects into mean (by the Luo et al., 2016 [16] transformation-based approach) and standard deviation (adopting the Wan et al., 2014 estimation [17]). Then, we estimated random effects pooled means and differences between means and their respective 95% confidence intervals (CIs) for the HAZ and BAZ. For the other nutritional measurements (fat and lean mass, weight-for-age, and waist circumference), the meta-analyses could not be estimated, considering the lack of combinable studies.

The heterogeneity and consistency of the studies were tested by the Cochran Q-test and quantified by the I^2^ test [12, 18]. The heterogeneity between the studies varies from 0 to 100%, and values close to zero suggest that there is no heterogeneity, considering that the dispersion can be attributed to random error; close to 25% indicates low heterogeneity; > 50% indicates moderate heterogeneity; and > 75% indicates high heterogeneity [12, 18].

Considering the small number of studies included in the meta-analysis, we could not conduct meta-regression and subgroup analysis to investigate the causes of the heterogeneity. For the same reason, publication bias was not evaluated by visual inspection of the funnel plot and calculating the Egger test [18]. Analyses were performed in Stata for Mac, version 16.0.

## Results

### Search results

**Fig. 1:**
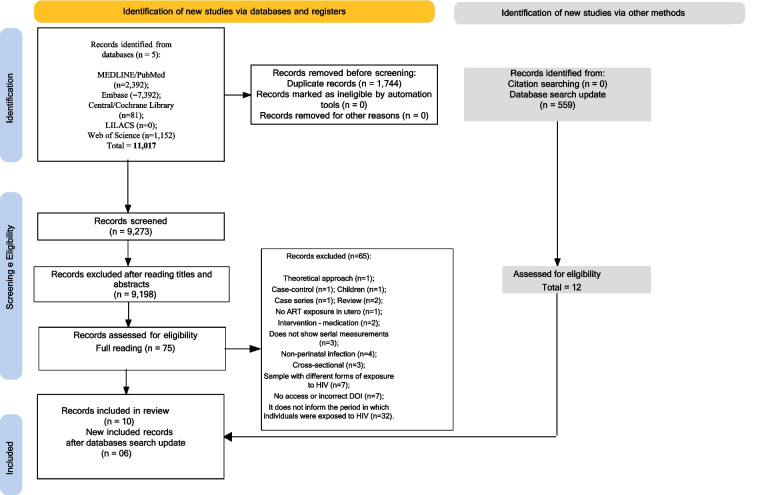
PRISMA flowchart of the search strategy and screening process

Our search strategy retrieved 11,017 documents. After excluding 1,744 duplicates, we analyzed 9,273 titles and abstracts. Full-text articles for the remaining 87 records were retrieved, of which 68 were excluded (12 were either retracted or could not be located due to a lack of DOI numbers). Of the 19 studies initially included for extraction, 9 were excluded during full-text review: 2 involved participants with HIV acquired through transfusion; 1 included a sample that was not exposed to HIV; 2 were case–control studies; 1 involved a sample not exposed to ART in utero; one included individuals not perinatally HIV-infected; and two provided no information on how the participants acquired HIV. The PRISMA flowchart illustrating the screening process is presented in Fig. 1.

The search update was conducted on June 3, 2024, covering the period from October 2022 to May 2024. During this time, 500 articles were found in Embase, 40 in PubMed, none in the Cochrane Central Library, none in LILACS, and 19 in Web of Science. In total, 559 studies were retrieved from the database update. During the title and abstract analysis, 12 publications were selected, but 6 of these did not meet the inclusion criteria upon full-text review. Consequently, six new publications were identified, two of which were different articles from the same study already included in the previous search. Therefore, this systematic review ultimately included 16 publications from 14 studies.

The 14 studies selected to compose this systematic review included: Anderson, Muloiwa and Davies, 2019 [19]; Bakeera-Kitaka et al., 2015 [20]; Boettiger et al., 2016 [21]; Bunupuradah et al., 2016 [22]; Buonora et al., 2008 [23]; Crichton et al., 2019 [24]; Desai, Mullen and Mathur, 2008 [25]; Dirajlal-Fargo et al., 2022 [26]; Fabiano et al., 2013 [27]; Foster et al., 2023 [28]; Mwambenu et al., 2022 [29]; Rehman et al., 2023 [30]; Rose et al., 2023 [31]; and three publications from the same study: Slogrove, Judd and Leroy, 2016 [32]; Jesson et al., 2022 [33], and Crichton et al., 2023 [34].

### Study and patient characteristics

The main characteristics of the included studies are summarized in Table 1. All studies had cohort designs and were published between 2008 and 2023. Four studies (six publications) were conducted multicenterally, involving two or more countries [21, 22, 24, 32]. Three studies were conducted in South Africa [19, 29, 31], while one each was conducted in the United States [25], Brazil [23], Uganda [20], Italy [27], the USA and Puerto Rico [26], England [28], and Zimbabwe and Malawi [30].

**Table 1:** Characteristics of the studies included in the systematic review

Author, year	Country	Population	Year of data collection	Sample	Age (years)		Follow-up times	HIV + sample?	Antiretroviral use (time of use)	Used antiretroviral	Outcomes & Instruments
					**Age range**	**Average or median**					
Anderson, Muloiwa Davies, 2018 [19]	South Africa	Adolescents and young adults	2002—2015	127 (62 men; 65 women)	10–22	Median = 15.1 (13 – 17.7)	12 years	Yes	Yes (10 years)	Nevirapine; nucleoside reverse transcriptase inhibitor; lopinavir; ritonavir; efavirenz; atazanavir	Weight and height
Bakeera-Kitaka et al., 2008 [20]	Uganda	Adolescents	2004—2006	118 (42 men; 76 women)	10–19	Average = 13.6	2 years	Yes	Yes	Zidovudina or estavudina, lamivudina combined with nevirapina or efavirenzFour patients were on a protease inhibitor-based regimen with lopinavir/ritonavir, in combination with AZT or D4T, plus 3TC	Weight, height and body mass index
Boettiger et al., 2016^a^ [21]	Multicentric	Adolescents	2003	534 (234 men; 300 women)	10–19	Median = 11.8 (10,7 – 13.2)	6 years	Yes	Yes	Lamivudine, zidovudine, efavirenz, stavudine, nevirapine, tenofovir	Weight and height
Bunupuradah et al., 2016^b^ [22]	Multicentric	Adolescents	2003	273 (109 men; 164 women)	11–18	-	7 years	Yes	Yes (7,3 years)	Nevirapine, efavirenz and ritonavir-boosted lopinavir. Nucleoside reverse transcriptase inhibitors were lamivudine, zidovudine and stavudine	Weight and height
Buonora et al., 2008 [23]	Brazil	Adolescents	2003	108 (47 men; 61 women)	10,5–19,5	Average = 12.7	8 years	Yes	Yes	-	Weight and length
Crichton et al., 2019^c^ [24]	Multicentric	Adolescents	2016—2017	463	10–16	Average = 1.66 ± 8.7 (men); 1.58 ± 6.9 (women)	9.1 years	Yes	Yes (9,1 years)	-	Weight, height and body mass index
Crichton et al., 2023^d^ [34]	Multicentric	Children and Adolescents	1994—2015	4.723 (1493 men; 3230 women)	07–15	Median (girls) = 13.9 (12.8–15.2) Median (boys) = 15.3 (14.6–16.4)	21 years	Yes	Yes (-)	Non-nucleoside reverse transcriptase inhibitors; nucleoside/nucleotide reverse transcriptase inhibitors; boosted protease inhibitor	Weight, height and body mass index
Desai, Mullen and Mathur, 2008 [25]	USA	Adolescents	1999—2001	35	10–15	-	-	Yes	Yes (2,4 years)	Protease inhibitors	Weight, height, body mass index and Bioimpedance (pro logic Omron HBF301 Healthcare, Inc. Vernon Hills, IL)
Dirajlal-Fargo et al., 2022 [26]	USA and Puerto Rico	Children and Adolescents	2007—2013	261 (132 men; 129 women)	07–19	Median = 12.25 (9,72 – 14.20)	2 years	Yes	Yes (-)	Protease inhibitors; nucleoside reverse transcriptase inhibitors; non-nucleoside reverse transcriptase inhibitors	Weight, height, and whole-body DXA with Lunar (General Electric Health Care) or Hologic (Hologic Inc) scanner
Fabiano et al., 2013 [27]	Italy	Adolescents and young adults	2004—2012	24	12–20	-	8 years	Yes	Yes (8 years)	Tenofovir; efavirenz	Weight, height, and dual-energy X-ray absorptiometry (DXA) scanner (Lunar Prodigy, GE-Lunar Radiation Corporation, WI, USA)
Foster et al., 2023 [28]	England	Adolescents and young adults	-	85 (49 men; 36 women)	15–24	Median = 22 (19.0–24.0)	2.2 years	Yes	Yes (-)	Tenofovir; alafenamida	Total body dual-energy X-ray absorptiometry (DXA), transient elastography of the liver (fibroscan)
Jesson et al., 2022^e^ [35]	Multicentric	Adolescents	1994—2015	20,939 (10.512 men; 10.427 women)	10–19	-	21 years	Yes	Yes ( -)	-	Weight, height and body mass index
Mwambenu et al., 2022 [29]	South Africa	Adolescents	2003—2016	288 (149 men; 139 women)	13–18	Median = 15.8 (14.2 – 17.6)	5 years	Yes	Yes (9 years)	Children > 3 years and 10 kg started on a nonnucleoside reverse transcriptase inhibitor	Weight, height and body mass index
Slogrove, Judd e Leroy, 2016^f^ [32]	Multicentric	Adolescents	1994—2015	37.614 (18.591 men; 19.023 women)	11–14	Median = 12.4 (11.0 – 14.4)	10 years	Yes	Yes	-	Weight, height and body mass index
Rehman et al., 2023 [30]	Zimbabwe and Malawi	Children and Adolescents	2018—2021	303 (152 men; 151 women)	06–19	Average = 12.5 (SD = 2.5)	1 year	Yes and No	Yes (2 years)	-	Dual-energy X-ray absorptiometry (DXA) total body scans under standard conditions using Hologic QDR Wi densitometer (Hologic Inc., Bedford, MA, USA)
Rose et al., 2023 [31]	South Africa	Adolescents	Recruitment: 2019—2021 Assessment: 2020—2022	68 (26 men; 42 women)	10–17	Median = 13.5 (12.5 – 14.4)	2 years	Yes	Yes (-)	Abacavir; Zidovudina; Tenofovir disoproxil fumarato; Efavirenz; Nevirapina; Lopinavir/ritonavir; Atazanavir/ritonavir; Dolutegravir	Weight, height, waist circumference, hip circumference and body mass index

^a^Cambodia, India, Indonesia, Malaysia, Thailand and Vietnam

^b^Cambodia, India, Malaysia, Vietnam, Thailand

^c^Thailand, United Kingdon/Irland

^d^Countries from all continents

^e^Countries from all continents

^f^Countries from all continents

Considering the populations included in the publications of this systematic review, ten studies focused solely on adolescents (*n* = 39,501) [20–25, 29, 31–33]; three articles included both children and adolescents (*n* = 5,287) [26, 30, 34]; and three publications evaluated adolescents and young adults (*n* = 236) [19, 27, 28]. Regarding exposure, all studies included perinatally HIV-infected individuals, except for the study conducted by Rehman et al. [30], which included both perinatally infected and uninfected individuals in its sample.

In total, 40,573 volunteers were evaluated. Among the thirteen publications that provided information by sex, 20,844 were women (51.4%). For the studies that focused solely on adolescents [20–25, 29, 31–33], the average follow-up time was 5.9 years. Among the studies that included adolescents and young adults, Anderson's study had a total follow-up time of 12 years [19], Fabiano et al. [27] followed participants for 8 years, and Foster et al. [28] had a follow-up period of 2.2 years. The follow-up periods for the studies including children and adolescents were 2 years for Dirajlal-Fargo et al. [26] and 1 year for Rehman et al. [30].

## Results of the studies

### Weight-for-age

Four studies evaluated the mean/median weight-for-age z-score (WAZ), following the classification recommended by the World Health Organization [36], while one study assessed the mean/median weight z-score in perinatally HIV-infected adolescents. Boettiger et al. (2016) [21], Mwambenu et al. (2022) [29], Anderson, Muloiwa, and Davies (2019) [19], and Bakeera-Kitaka et al. (2015) [20] calculated the median WAZ and reported values of −2.6 (IQR: −3.6; −1.4), −1.5 (IQR: −2.5; −0.8), −1.97 (IQR: −3.23; −0.66), and −2.61 (IQR: −3.93; −1.67) at the beginning of their studies, respectively. Additionally, Bakeera-Kitaka et al. (2015) [20] calculated the median WAZ at follow-up (one year later) and found a value of −1.26 (IQR: −2.5; −0.4). Buonora et al. (2008) [23] identified a mean weight z-score of −0.84 (SD: 1.42) at the start of the study and −1.15 (SD: 1.32) at follow-up (8 years later).

### Underweight

Additionally, Mwambenu et al. (2022) [19] reported a prevalence of underweight (WAZ ≤ −2) of 36.7% at the beginning of their study, while Buonora et al. (2008) [23] identified a prevalence of weight z-scores ≤ −2 of 17.6% at baseline and 29.2% at follow-up. Rehman et al. (2023) reported a relative risk of underweight of 1.75 (95% CI 0.98–3.15) in perinatally infected children and adolescents compared to the uninfected group. They also found a relative risk of 8.26 (95% CI 3.92–17.4) for underweight and stunting, and 28.8 (95% CI 3.67–226.8) for very underweight and stunting when comparing perinatally infected individuals to those who were uninfected.

### Height-for-age

Ten publications assessed the height-for-age z-score (HAZ) mean or median at the beginning of the study and during adolescence or young adulthood, as well as the mean difference by year in perinatally HIV-infected individuals [19–24, 29, 32–34]. All studies utilized the classification recommended by the World Health Organization [36] for the HAZ parameter.

The median HAZ identified by Crichton et al. (2019) [24] was −1.2 (IQR: −2.3, −0.2) at the beginning of the study, with a median age of 6.4 years (IQR: 2.8, 9.0). At age 16, the mean heights for boys and girls were 166 cm (SD: 8.7) and 158 cm (SD: 6.9), respectively. Boettiger et al. (2016) [21], Bunupuradah et al. (2016) [22], Slogrove, Judd, & Leroy (2016) [32], Anderson, Muloiwa, and Davies (2019) [19], Bakeera-Kitaka et al. (2015) [20], and Mwambenu et al. (2022) [29] reported median HAZ values of −2.3 (IQR: −3.6, −1.4), −2.2 (IQR: −3.2, −1.4), −1.54 (IQR: −2.06, −0.72), −2.92 (IQR: −4.09, −1.95), −2.69 (IQR: −3.57, −1.78), and −2.2 (IQR: −3.1, −1.3), respectively, at the start of the study. After the follow-up, the median HAZ values were −1.6 (IQR not presented), −1.5 (IQR: −2.2, −0.9), −1.60 (IQR: −2.46, −0.73), −1.52 (IQR: −2.22, −0.79), −2.58 (IQR: −3.3, −1.6), and −1.1 (IQR: −1.8, −0.6), respectively. Buonora et al. (2008) [23] calculated a mean HAZ of −0.94 (SD: 1.23) at the beginning of the study and −1.20 (SD: 1.18) at follow-up.

Crichton et al. (2023) [34] reported median HAZ values at the beginning of the study of −2.0 (IQR: −2.9 to −1.4) for girls and −1.8 (IQR: −2.8 to −1.0) for boys. The authors did not assess HAZ values after the follow-up. Jesson et al. (2022) [33] calculated a mean HAZ difference per year of −0.014 (SD: 0.006) for girls with moderate wasting and −0.052 (SD: 0.012) for those with severe wasting. For boys, the mean difference per year was −0.007 (SD: 0.001) for those with moderate wasting and −0.011 (SD: 0.002) for those with severe wasting.

### BMI

#### Studies including perinatally HIV-infected individuals

Nine studies that included only perinatally HIV-infected individuals evaluated BMI. Four of these studies assessed the BMI-for-age z-score (BAZ) in adolescents, three evaluated BMI in both adolescents and adults, and two examined this parameter in children and adolescents. In adolescents, Mwambenu et al. (2022) [29] calculated the prevalence of BAZ < −2 (according to WHO classification) [36] and found 9.1% at the beginning of the study and 6.7% at follow-up. Additionally, Bakeera-Kitaka et al. (2015) [20], Slogrove, Judd, and Leroy (2016) [32], Mwambenu et al. (2022) [29], and Rose et al. (2023) [31] calculated the median values of BAZ in their populations, finding −1.61 (IQR: −2.49; −0.81), −0.54 (IQR: −1.26; 0.13), −0.2 (IQR: −1.0; 0.6), and 0.04 (IQR: −0.81; 0.81), respectively, at the beginning of the study; and −0.68 (IQR: −1.3; 0.1), −0.68 (IQR: −1.46; 0.09), −0.6 (IQR: −1.4; 0.1), and 0.05 (IQR: −0.72; 1.03), respectively, after the follow-up period.

Considering the studies that evaluated the BMI parameter in children and adolescents, Dirajlal-Fargo et al. (2022) [26] identified a median BAZ of 0.2 (IQR: −0.4; 1.2) at the beginning of the study and 0.2 (IQR: −0.4; 1.1) after 2 years of follow-up. Crichton et al. (2023) [34] estimated median BAZ values of −0.6 (IQR: −1.5; 0.1) for girls and −0.7 (IQR: −1.6; 0.1) for boys at the beginning of the study, and 0.004 (IQR: 0.001; 0.008) for girls and 1.618 (IQR: 0.680; 2.557) for boys after the follow-up.

For studies including adolescents and young adults, Anderson, Muloiwa and Davies (2019) [19] calculated prevalence of BAZ < −2 and found 13.3% at the beginning of the study and 5.5% after the follow-up. Additionally, Anderson, Muloiwa and Davies (2019) [19] calculated the median of the BAZ in their population and found 0.2 (IQR: −0.78; 1.25) at the beginning of the study; and −0.16 after the follow-up period. Fabiano et al. (2013) [27] identified a BMI mean of 18.9 kg/m2 (CI95% 17.9–19.8) at the beginning of the study and 21 kg/m2 (CI95% 19.8–22.3) at the follow-up. Foster et al. (2023) [28] calculated the BMI mean at the beginning of the study and identified a value of 25,7 kg/m2 (SD: 5,4). They did not estimate the mean value after the follow-up. Instead, they reported a BAZ mean value of 0,6 (SD: 2,7) after the follow-up period.

For studies including adolescents and young adults, Anderson, Muloiwa, and Davies (2019) [19] calculated the prevalence of BAZ < −2 and found it to be 13.3% at the beginning of the study and 5.5% after the follow-up. They also reported a median BAZ of 0.2 (IQR: −0.78; 1.25) at the start of the study, which decreased to −0.16 after the follow-up period. Fabiano et al. (2013) [27] identified a mean BMI of 18.9 kg/m^2^ (95% CI: 17.9–19.8) at the beginning of the study and 21 kg/m^2^ (95% CI: 19.8–22.3) at follow-up. Foster et al. (2023) [28] recorded a mean BMI of 25.7 kg/m^2^ (SD: 5.4) at the beginning of the study, but they did not estimate the mean value after follow-up. Instead, they reported a mean BAZ of 0.6 (SD: 2.7) after the follow-up period.

### Waist circumference

Three studies evaluated waist circumference (WC). Fabiano et al. (2013) [27] and Rose et al. (2023) [31] assessed perinatally HIV-infected adolescents in their samples, identifying median values of 68 cm (IQR: 65–70) and 64.8 cm (IQR: 61.5–73.8) at the beginning of the study, respectively. At follow-up, the median values were 74 cm (IQR: 67–80) and 71.4 cm (IQR: 65.6–79.3), respectively.

### Fat mass

Three studies evaluated fat mass in perinatally HIV-infected individuals. Two of these studies focused on perinatally HIV-infected children and adolescents. Desai, Mullen, and Mathur (2008) [25] assessed total body fat using bioelectrical impedance, reporting body fat percentages (%BF) of 34% (range: 23.9 to 45; SD = 7.4) at the beginning of the study and 38% (range: 31.3 to 47.3; SD = 5.1) after 18 months of follow-up. Dirajlal-Fargo et al. (2022) [26] identified a median %BF of 21.6% (IQR: 14.9 – 29.2) at the start of the study and 21.5% (IQR: 13.7–30.3) after the follow-up period. One study focused on the adolescents and young adult age group. Fabiano et al. (2013) [27] evaluated body composition in perinatally HIV-infected adolescents and young adults using dual-energy X-ray absorptiometry (DXA). They found a prevalence of high total %BF of 16.2% (95% CI: 13.4 to 19.0) at the beginning of the study, with a linear increase of 0.6% (0.2 to 1.0) per year (*p* = 0.005). Arm fat percentage was 8.1% (range: 7.5 to 8.6) at baseline and remained stable at follow-up (*p* = 0.5). Leg fat was 42.8% (range: 39.6 to 45.8) at the beginning of the study and decreased linearly by 1.1% (1.5 to 0.7) per year (*p* < 0.001) at follow-up. Trunk fat percentage was 49.1% (range: 46.2 to 52.1) at the start and increased linearly by 1.2% (0.6 to 1.6) per year (*p* < 0.001).

### Meta-analysis

**Fig. 2:**
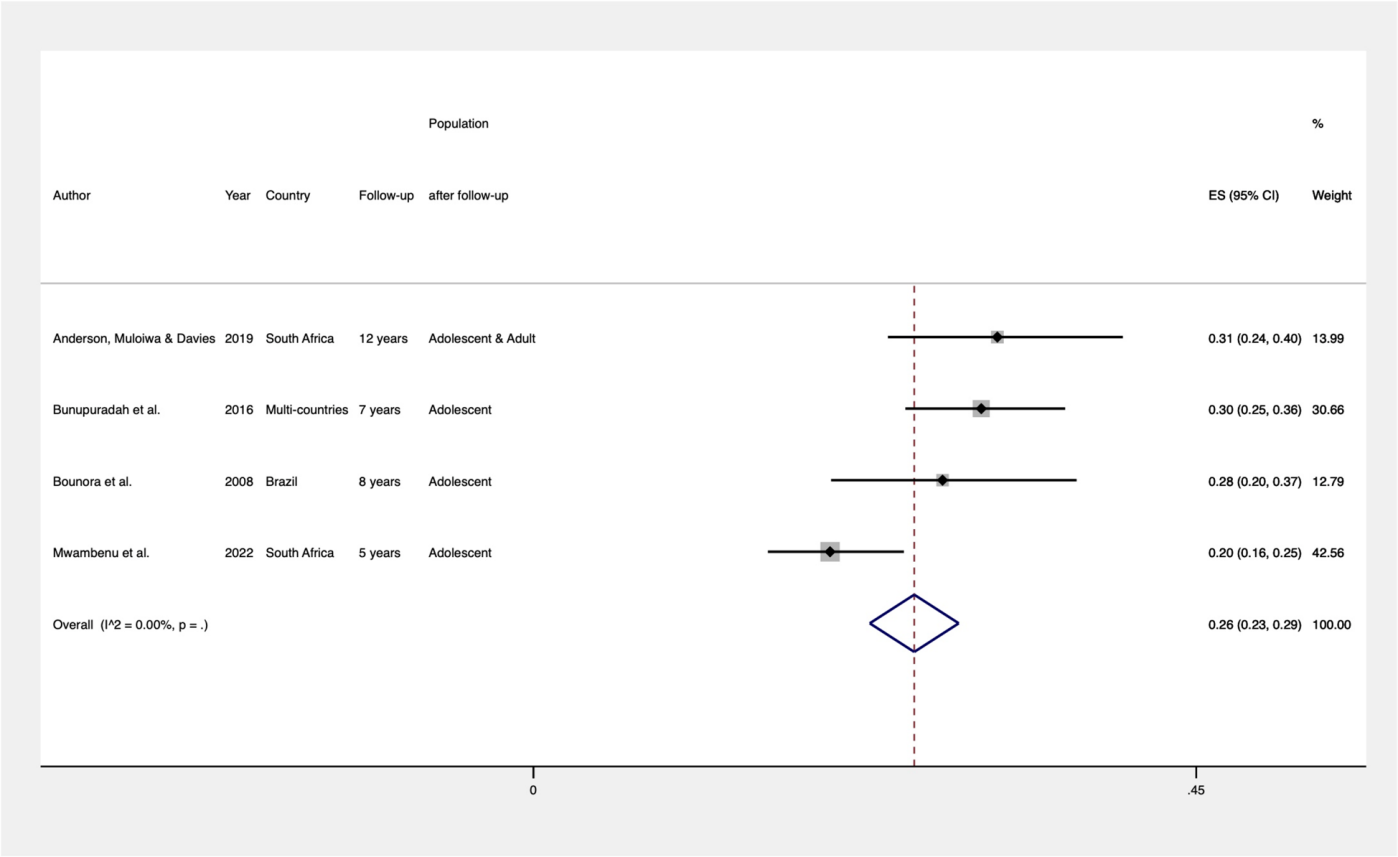
Pooled prevalence of stunting (HAZ < −2) in perinatally HIV-infected individuals

**Fig. 3:**
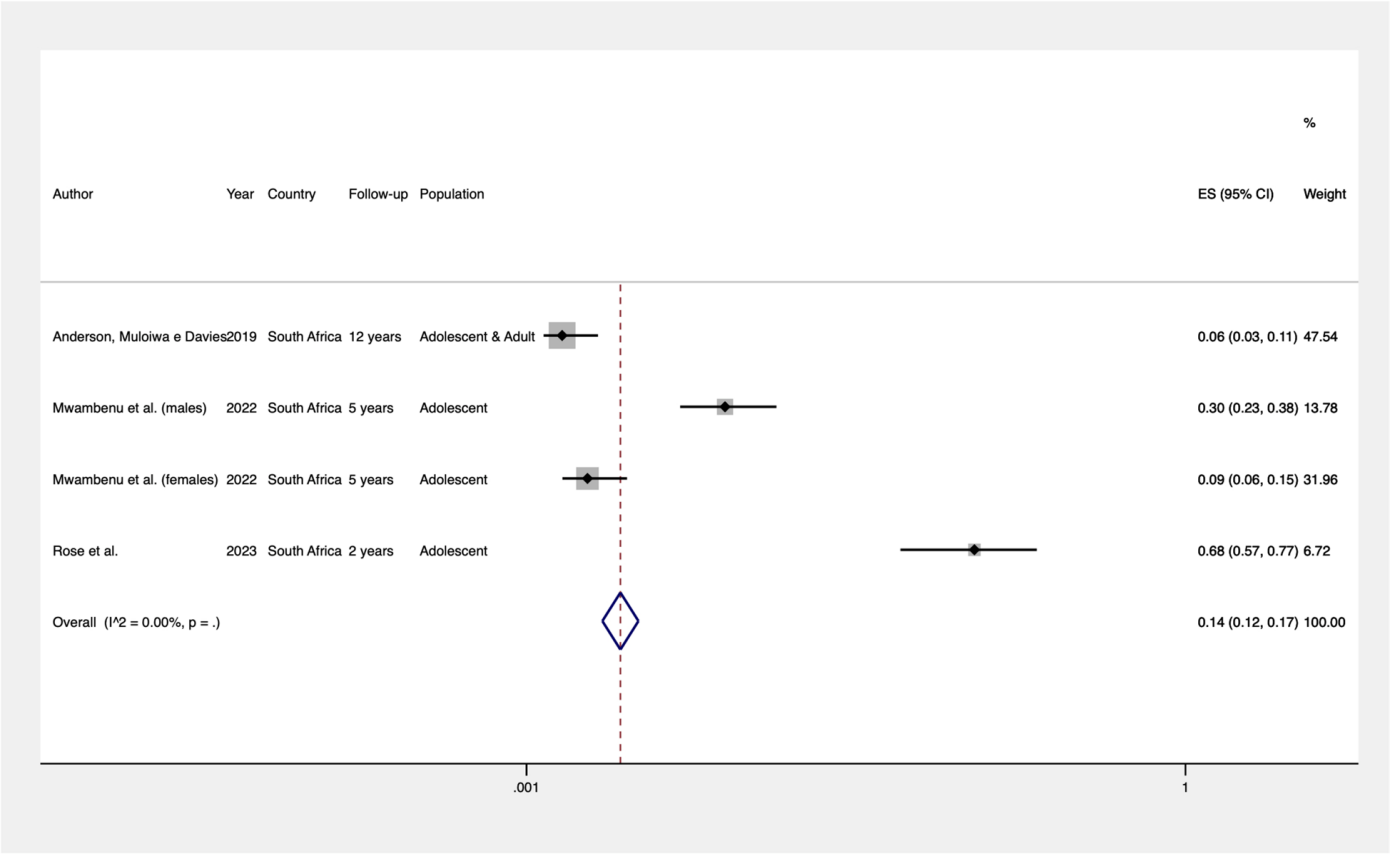
Pooled prevalence of underweight (BAZ < −2) in perinatally HIV-infected individuals

The prevalence of stunting and underweight was calculated for the combinable studies and is presented in Figs. 2 and 3, respectively. Studies were deemed combinable if they included a similar population (perinatally HIV-infected adolescents and young adults) and provided either a precalculated prevalence rate or the number of adolescents with low HAZ or BAZ alongside the total sample size. All studies utilized the HAZ and BAZ classifications recommended by the World Health Organization [36]. As a result, four studies were included in the meta-analysis to determine the global prevalence of stunting among perinatally HIV-infected adolescents and young adults. We found that 26% (95% CI: 0.23–0.29) of these individuals were stunted (Fig. 2). For BAZ, three studies were combined, revealing a global prevalence of 14% (95% CI: 0.12–0.17) for underweight in perinatally HIV-infected adolescents and young adults (Fig. 3). For other nutritional outcomes, we could not estimate summary prevalence due to a lack of combinable studies and/or available information.

**Fig. 4:**
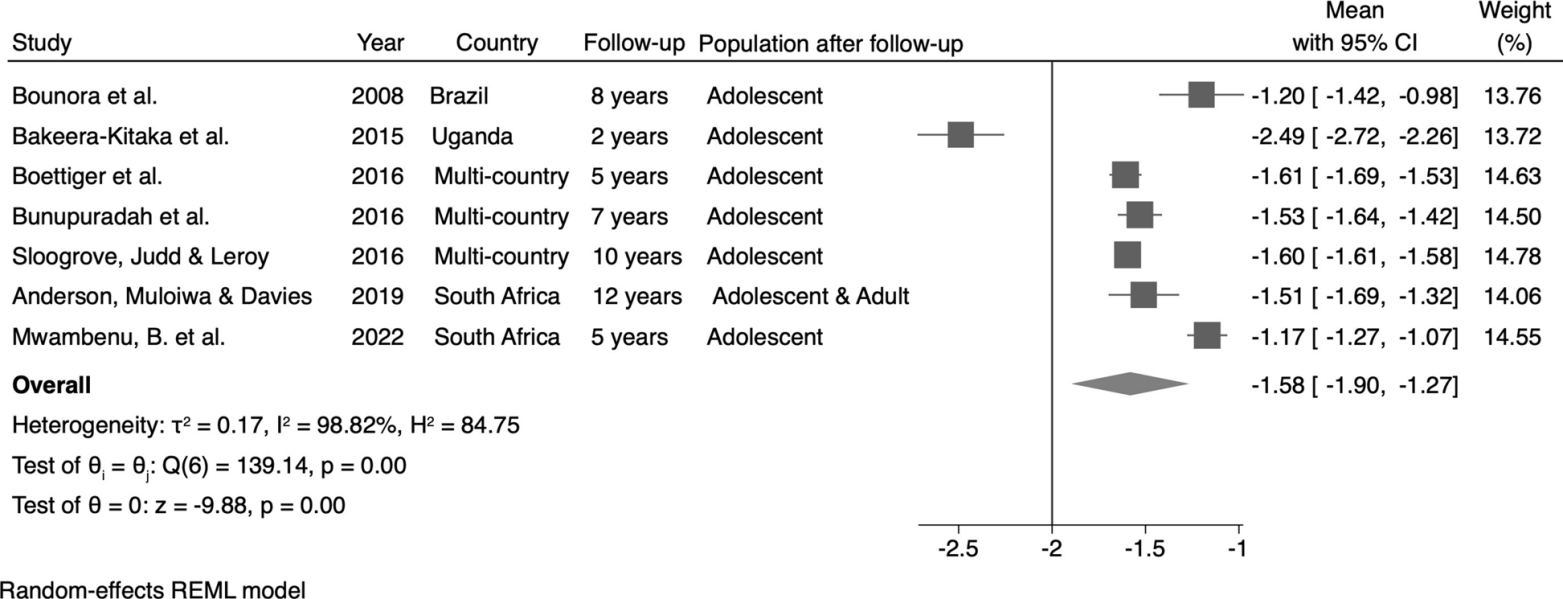
Pooled HAZ mean value for perinatally HIV-infected individuals

**Fig. 5:**
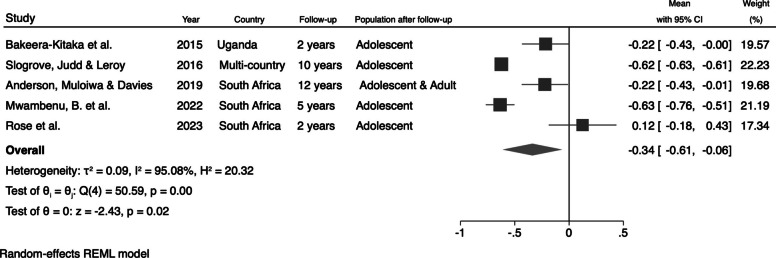
Pooled BAZ mean value for perinatally HIV-infected individuals

For the studies that evaluated the mean values of HAZ and BAZ, we also calculated the pooled estimates for these anthropometric parameters, focusing on the combinable studies. The mean HAZ for perinatally HIV-infected adolescents and young adults was −1.58 (95% CI: −1.90; −1.27) (Fig. 4). For BAZ, the pooled mean value for perinatally HIV-infected individuals was −0.34 (95% CI: −0.61; −0.06) (Fig. 5).

**Fig. 6:**
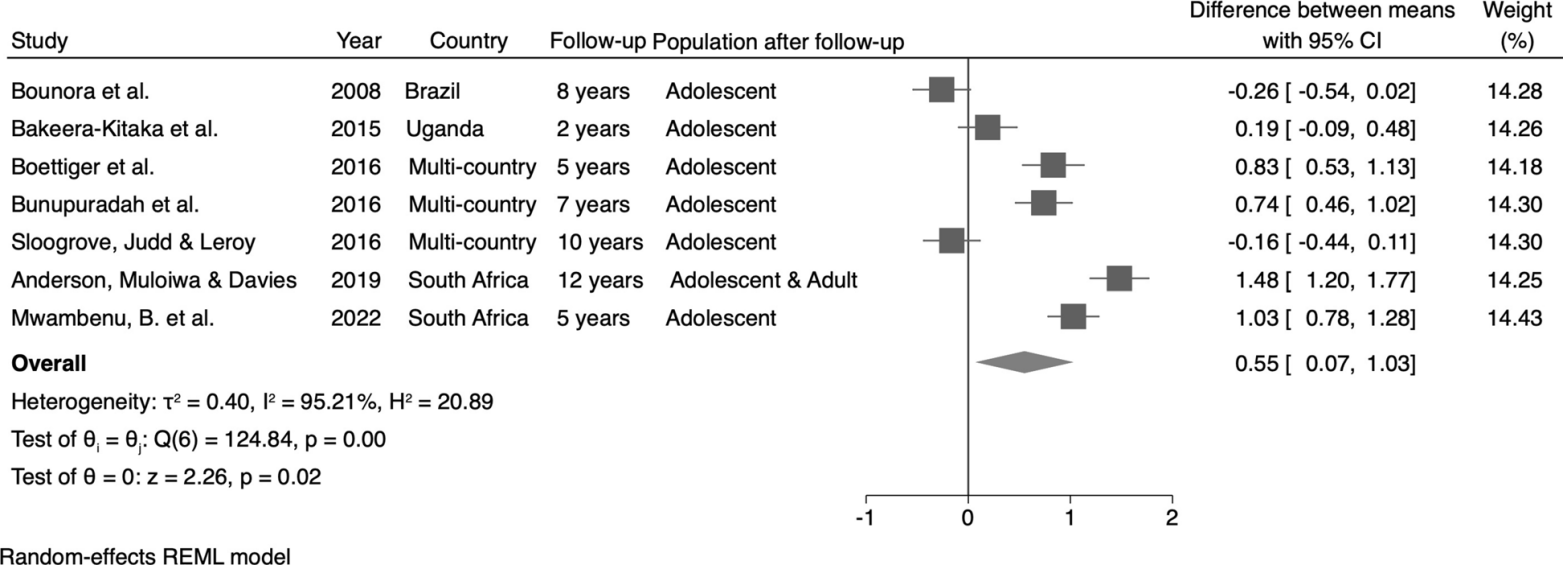
Pooled changes in the mean values of HAZ in perinatally HIV-infected individuals

**Fig. 7:**
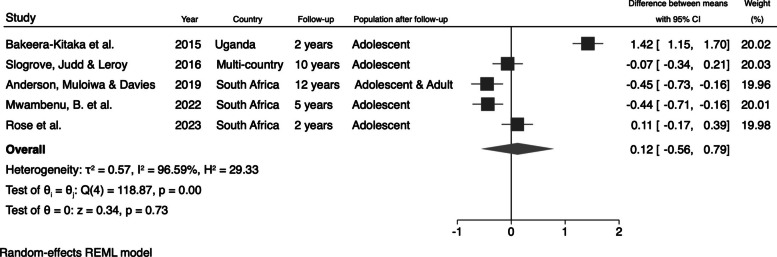
Pooled changes in the mean values of BAZ in perinatally HIV-infected individuals

We also calculated the changes in the mean values of HAZ and BAZ after the follow-up period by subtracting the initial mean from the final mean. We identified an increase of 0.55 (95% CI: 0.07; 1.03) in the mean HAZ (Fig. 6) and an increase of 0.12 (95% CI: −0.56; 0.79) in the mean BAZ (Fig. 7) for perinatally HIV-infected individuals after the follow-up period.

### Quality assessment

Considering the 14 studies (16 publications) included in this systematic review, 10 presented a moderate risk of bias, while 4 showed a high risk of bias. The factors contributing to this increased risk included the absence of a comparison group, a lack of clarity or declaration regarding strategies to address confounding factors in the analyses, and insufficient detail on follow-up, including whether participants were fully followed up and, if not, whether the studies reported and explored the reasons for loss to follow-up. Additionally, there was low clarity regarding strategies to address incomplete follow-up. More details on the risk of bias are provided in Table 2.

**Table 2:** Results of the risk of bias analysis for the included studies

Questions	Anderson, Muloiwa and Davies, 2019	Bakeera-Kitaka et al., 2015	Boettiger et al., 2016 [21]	Bunupuradah et al., 2016 [22]	Bounora et al., 2008 [23]	Crichton et al., 2019 [24]	Desai, Mullen and Mathur, 2008 [25]	Dirajlal-Fargo et al., 2022 [26]	Fabiano et al., 2013 [27]	Foster et al., 2023 [28]	Mwambenu et al., 2022 [29]	Rehman et al., 2023 [30]	Rose et al., 2023 [31]	Sloogrove, Judd and Leroy, 2016^a^ [32]
Were the two groups similar and recruited from the same population?	NA	NA	NA	NA	NA	NA	NA	NA	NA	NA	NA	Y	NA	NA
Were the exposures measured similarly to assign people	NA	NA	NA	NA	NA	NA	NA	NA	NA	NA	NA	Y	NA	NA
To both exposed and unexposed groups?	NA	NA	NA	NA	NA	NA	NA	NA	NA	NA	NA	Y	NA	NA
Was the exposure measured in a valid and reliable way?	Y	Y	Y	Y	Y	Y	Y	Y	Y	Y	Y	Y	Y	Y
Were confounding factors identified?	Y	U	U	U	NA	U	NA	Y	Y	U	NA	U	Y	Y
Were strategies to deal with confounding factors stated?	Y	N	Y	U	NA	U	NA	Y	Y	U	NA	U	Y	Y
Were the groups/participants free of the outcome at the start of the study (or at the moment of exposure)?	Y	Y	Y	Y	Y	Y	Y	Y	Y	Y	Y	Y	Y	Y
Were the outcomes measured in a valid and reliable way?	Y	Y	U	Y	Y	Y	Y	Y	Y	Y	Y	Y	Y	Y
Was the follow up time reported and sufficient to be long enough for outcomes to occur?	Y	Y	Y	Y	Y	Y	Y	Y	Y	Y	Y	Y	Y	Y
Was follow up complete, and if not, were the reasons to loss to follow up described and explored?	Y	Y	N	Y	U	Y	U	N	Y	Y	U	N	Y	N
Were strategies to address incomplete follow up utilized?	NA	Y	U	NA	U	NA	U	U	NA	Y	U	U	NA	Y
Was appropriate statistical analysis used?	Y	Y	U	Y	Y	Y	Y	Y	Y	Y	Y	Y	Y	Y
**Classification**	Moderate risk of bias	Moderate risk of bias	High risk of bias	Moderate risk of bias	High risk of bias	Moderate risk of bias	High risk of bias	Moderate risk of bias	Moderate risk of bias	Moderate risk of bias	High risk of bias	Moderate risk of bias	Moderate risk of bias	Moderate risk of bias

*Abbreviations*: *Y* Yes, *N* Not, *U* Unclear, *NA* Not applicable

^a^The articles by Crichton et al. (2023), Sloogrove, Judd, and Leroy (2016), and Jesson et al. (2022) are different publications using data from the same study; therefore, the bias risk analysis was conducted for the overall study rather than for the individual publications

### Difference between the protocol and the systematic review

Some methods in the protocol could not be applied due to insufficient data or a lack of information from the articles included in the review. We aimed to undertake one meta-analysis for each outcome variable (HAZ, BMI, BAZ, waist circumference and lean mass). However, considering the lack of information from the articles included in this review, we only could undertake meta-analysis for the indicators BMI-for-age (BAZ) and Height-for-age (HAZ).

## Discussion

In this study, we assessed the growth and body composition of perinatally HIV-infected adolescents and young adults and we observed that they continue to fail to thrive. Our results reveal significant rates of stunting and underweight. The meta-analysis indicated a prevalence of 26% for stunting and 14% for underweight among this population, highlighting the urgent need for targeted nutritional interventions. These findings are particularly concerning given the long-term health implications associated with stunting and underweight, which can adversely affect physical growth, cognitive development, and overall well-being. The use of World Health Organization classification standards for height-for-age (HAZ) and body mass index-for-age (BAZ) ensures the reliability of our results, allowing for meaningful comparisons across studies.

Additionally, the pooled mean values for height-for-age (HAZ) and body mass index-for-age (BAZ) reveal a troubling trend in the growth trajectories of these adolescents. The average HAZ of −1.58 indicates that perinatally HIV-infected individuals are significantly below the expected height for their age. Although there was a modest increase of 0.55 in HAZ during the follow-up period, this improvement highlights the persistent challenges this population faces in achieving optimal growth. In contrast, the slight increase in BAZ of 0.12 suggests some progress; however, it remains insufficient to fully address the broader issue of underweight, as indicated by the negative mean value still observed. Notably, studies reporting the BMI-for-age z-scores indicated mean values within the normal limits set by the World Health Organization. This suggests that while delayed weight gain is common among adolescents with perinatally acquired HIV, their overall body mass index is less adversely affected. This discrepancy emphasizes the complexity of growth patterns in this population, where height and weight trajectories may diverge, necessitating targeted nutritional strategies to ensure comprehensive health and development.

This systematic review contributes to the growing body of literature indicating that, despite improvements in weight, growth failures persist among perinatally HIV-infected adolescents and young adults. The growth failure observed in this study may be attributed to several underlying mechanisms that vary according to individual HIV status and the age at which antiretroviral therapy (ART) is initiated [29, 37, 38]. These mechanisms include, but are not limited to, the effects of the infection itself, virological and immunological control, opportunistic infections, endocrine alterations, nutrient intake [39], nutrient absorption [40], and psychosocial factors [41, 42]. Virologic control is closely linked to sustained growth; however, it is insufficient for reaching the growth averages of the general population [29, 35, 43].

Poor nutritional status significantly impacts the health of perinatally HIV-infected adolescents and young adults, exacerbating the challenges they face in managing their condition. Malnutrition, characterized by underweight and stunting, can weaken the immune system, making these individuals more susceptible to opportunistic infections and disease progression. Inadequate nutrient intake can also impair growth and development, further compromising physical health during critical periods of adolescence and young adulthood [44, 45]. Malnutrition has significant effects on mortality and morbidity among adults living with HIV [46]. As the degree of undernutrition became more severe, mortality rate also increased. Moreover, poor nutrition can affect the efficacy of ART, leading to suboptimal treatment outcomes and increased viral loads [47–50]. The resulting interplay between nutritional deficiencies and HIV-related complications can create a vicious cycle, where deteriorating health further limits access to adequate nutrition, ultimately hindering the overall well-being and quality of life for these individuals. Addressing nutritional needs is essential for improving health outcomes and promoting resilience in this vulnerable population.

Perinatally HIV-infected adolescents and young adults face a significant burden of morbidity that affects their physical, mental, and social well-being. Chronic health issues, including opportunistic infections, respiratory diseases, bone pathologies [51], and metabolic complications, are prevalent in this group, often exacerbated by factors such as inconsistent access to healthcare and treatment adherence [8, 9, 51, 52]. As such, addressing the comprehensive health needs of this population is vital for improving their overall health outcomes and enhancing their ability to thrive in various aspects of life.

Evidence has shown that HIV-exposed but uninfected (HEU) children have higher morbidity and mortality rates compared to those who are unexposed to HIV and antiretroviral therapy (ART) [32]. Desmonde and collaborators (2016) [53] reported growth impairments among this population. One potential explanation for the growth failure observed in HEU children is their increased susceptibility to infectious diseases throughout their lives [54]. However, this hypothesis requires further investigation, as other factors, including residual confounding, may also influence this relationship. For perinatally HIV-infected adolescents and young adults, the mortality rate remains a significant public health concern, severely affecting the health outcomes of this vulnerable population. Despite advancements in ART and improved access to healthcare, studies indicate that these individuals experience higher mortality rates than their HIV-uninfected peers. Contributing factors to this elevated mortality include late diagnosis, inadequate treatment adherence, and the long-term effects of chronic HIV infection, such as opportunistic infections and non-communicable diseases [52, 55]. Addressing these complex challenges is crucial for improving survival rates and overall quality of life for perinatally HIV-infected adolescents and young adults, underscoring the urgent need for targeted interventions and comprehensive support systems.

Our results emphasize the complex interplay between HIV infection and nutritional outcomes in adolescents and young adults. The observed prevalence rates and mean values serve as a call to action for public health policymakers and healthcare providers to develop comprehensive strategies that address the nutritional needs of this vulnerable population. Enhanced nutritional support, alongside regular monitoring of growth parameters, could mitigate the adverse effects of perinatal HIV infection on growth and development. Furthermore, this study highlights the necessity for further research to explore additional nutritional outcomes and the underlying factors contributing to these disparities, which are essential for tailoring effective interventions.

Ten of the 14 studies included in this systematic review showed moderate risk of bias, especially due to the absence of a comparison group, lack of clarity in identifying confounders in their analyses and which strategies were adopted to address confounding factors in the analyses undertaken. This is an important limitation, since the presence of confounders can generate spurious results. In addition, the lack of clarity regarding follow-up strategies to address losses and if they were identified, described and considered in the analysis was one of the limitations of these studies. Therefore, the results presented here should be interpreted with caution due to these methodological weaknesses identified in the aforementioned studies.

Despite all the methodological rigor applied in conducting this systematic review with meta-analysis, some limitations should be considered. The results of the meta-analysis showed high heterogeneity, and its causes could not be assessed through subgroup analysis or meta-regression due to the low number of studies included. However, this high heterogeneity was expected, considering the different settings of the studies, different age groups, follow-up time, ART usage duration, as well as the timing of outcome measurement assessment. In addition, the small number of studies and their limitations such as lack of comparison group have restricted the ability to draw consistent conclusions about the effect and magnitude of perinatally HIV-infection on anthropometric outcomes. The combinable studies of this review included only perinatally HIV-infected individuals, with descriptive results, which makes the comparison with the growth pattern of perinatally HIV-uninfected individuals unfeasible. More research is critically needed to better understand these relationships and contribute to informing prevention and intervention strategies to reduce low height in infected children.

## Conclusions

In conclusion, our study highlights the alarming growth and body composition issues faced by perinatally HIV-infected adolescents and young adults, with prevalence considerable rates of stunting and underweight, emphasizing the urgent need for targeted nutritional interventions. The average HAZ indicates that these individuals are significantly below the expected height for their age, despite a modest increase during the follow-up period, revealing persistent challenges in achieving optimal growth. While the slight improvement in BAZ suggests some progress, it remains inadequate to fully address underweight concerns. However, these findings should be interpreted with caution, considering the small number and the limitations of the studies included in the review. Further studies are needed to describe the effect of HIV on the anthropometry of this population in greater detail. This information is needed for planning targeted interventions to meet the special needs that perinatally HIV-infected individuals have in their lives.

## Supplementary Information

Supplementary Material 1. Search Strategy.

## Data Availability

The data that support the findings of this study are not openly available due to reasons of sensitivity and are available from the corresponding author upon reasonable request. Please contact the author PRFC (prfarias@ufba.br).

## Abbreviations

ART: Antiretroviral therapy
HIV: Human immunodeficiency virus
HEU: HIV-exposed uninfected
PHIV: Perinatally HIV-infected
HAZ: Height-for-age
BAZ: BMI-for-age
