# Supplementary material for: Growth and body composition of adolescents and young adults with perinatal HIV infection: a systematic review and meta-analysis
Source: BMC Public Health. 2025 Feb 21;25:717. doi: 10.1186/s12889-025-21838-w (PMC11843769; doi:10.1186/s12889-025-21838-w)
Supplement: Supplementary file 1 — Supplementary Material 1. Search Strategy. [file 12889_2025_21838_MOESM1_ESM.pdf]

Search Strategy

Date: Oct 19th, 2022

Update: Jun 24th, 2024

1.1 PubMed

("hiv"[MeSH Terms] OR "Human Immunodeficiency Virus"[All Fields] OR "immunodeficiency virus human"[All Fields] OR "immunodeficiency viruses human"[All Fields] OR "virus human immunodeficiency"[All Fields] OR "viruses human immunodeficiency"[All Fields] OR "Human Immunodeficiency Viruses"[All Fields] OR "HIV Infections"[MeSH Terms] OR "HIV Infection"[All Fields] OR "infection hiv"[All Fields] OR "infections hiv"[All Fields] OR "hiv 1"[MeSH Terms] OR "Human immunodeficiency virus 1"[All Fields] OR "HIV-I"[All Fields] OR "immunodeficiency virus type 1 human"[All Fields] OR "Human Immunodeficiency Virus Type 1"[All Fields] OR "hiv 2"[MeSH Terms] OR "immunodeficiency virus type 2 human"[All Fields] OR "Human Immunodeficiency Virus Type 2"[All Fields] OR "Human immunodeficiency virus 2"[All Fields] OR "Human T-Lymphotropic Virus Type IV"[All Fields] OR "anti retroviral agents"[MeSH Terms] OR "agents anti retroviral"[All Fields] OR "anti retroviral agents"[All Fields] OR "Antiretroviral Agents"[All Fields] OR "agents antiretroviral"[All Fields] OR "Antiretroviral Agent"[All Fields] OR "agent antiretroviral"[All Fields]) AND ("infant"[MeSH Terms] OR "Infants"[All Fields] OR "child"[MeSH Terms] OR "Children"[All Fields] OR "adolescent"[MeSH Terms] OR "Adolescents"[All Fields] OR "Adolescence"[All Fields] OR "Teens"[All Fields] OR "Teen"[All Fields] OR "Teenagers"[All Fields] OR "Teenager"[All Fields] OR "Youth"[All Fields] OR "Youths"[All Fields] OR "adolescents female"[All Fields] OR "adolescent female"[All Fields] OR "Female Adolescent"[All Fields] OR "Female Adolescents"[All Fields] OR "adolescents male"[All Fields] OR "adolescent male"[All Fields] OR "Male Adolescent"[All Fields] OR "Male Adolescents"[All Fields] OR "Young Adult"[MeSH Terms] OR "adult young"[All Fields] OR "adults young"[All Fields] OR "Young Adults"[All Fields]) AND ("Growth and Development"[MeSH Terms] OR "Development and Growth"[All Fields] OR "growth"[MeSH Terms] OR "Body Size"[MeSH Terms] OR "Body Sizes"[All Fields] OR "size body"[All Fields] OR "sizes body"[All Fields] OR "Body Composition"[MeSH Terms] OR "Body Compositions"[All Fields] OR "composition body"[All Fields] OR "compositions

body"[All Fields] OR "Body Fat Distribution"[MeSH Terms] OR "distribution body fat"[All Fields] OR "fat distribution body"[All Fields] OR "Body Fat Patterning"[All Fields] OR "obesity"[MeSH Terms] OR "overweight"[MeSH Terms])

2,392 results

1.2. Embase

#1

'human immunodeficiency virus'/exp OR 'human immunodeficiency virus'/syn OR 'human immunodeficiency virus infection'/exp OR 'human immunodeficiency virus infection'/syn OR 'human immunodeficiency virus 1'/exp OR 'human immunodeficiency virus 1'/syn OR 'human immunodeficiency virus 2'/exp OR 'human immunodeficiency virus 2'/syn OR 'antiretrovirus agent'/exp OR 'antiretrovirus agent'/syn

#2

'infant'/exp OR 'infant'/syn OR 'child'/exp OR 'child'/syn OR 'adolescent'/exp OR 'adolescent'/syn OR 'young adult'/exp OR 'young adult'/syn

#3

'growth'/exp OR 'growth'/syn OR 'body size'/exp OR 'body size'/syn OR 'body composition'/exp OR 'body composition'/syn OR 'body fat distribution'/exp OR 'body fat distribution'/syn OR 'obesity'/exp OR 'obesity'/syn

#1 AND #2 AND #3

Results: 7.392

1.3. Cochrane Library

ID Search Hits

#1 MeSH descriptor: [HIV] explode all trees

#2 MeSH descriptor: [HIV-1] explode all trees

#3 MeSH descriptor: [HIV-2] explode all trees

#4 MeSH descriptor: [Anti-Retroviral Agents] explode all trees

#5 #1 OR #2 OR #3 OR #4

Results: 5924

#6 MeSH descriptor: [Infant] explode all trees

#7 MeSH descriptor: [Child] explode all trees

#8 MeSH descriptor: [Adolescent] explode all trees

#9 MeSH descriptor: [Young Adult] explode all trees

#10 #6 OR #7 OR #8 OR #9

Results: 196907

#11 MeSH descriptor: [Growth] explode all trees

#12 MeSH descriptor: [Body Size] explode all trees

#13 MeSH descriptor: [Body Composition] explode all trees

#14 MeSH descriptor: [Body Fat Distribution] explode all trees

#15 MeSH descriptor: [Obesity] explode all trees

#16 MeSH descriptor: [Overweight] explode all trees

#17 #11 OR #12 OR #13 OR #14 OR #15 OR #16

Results: 37.506

#18 #5 AND #10 AND #17

Results: 81

1.4. LILACS

#1 "HIV" OR "Vírus da Imunodeficiência Humana" OR "Vírus de Imunodeficiência Humana" OR "VIH" OR "HIV-1 " OR "VIH-1" OR "Vírus 1 da Imunodeficiência Humana" OR "HIV-2" OR "Vírus 2 da Imunodeficiência Humana" OR "Antirretrovirais" OR Agente Antirretroviral" OR "Agentes Antirretrovirais" OR "Antirretroviral" OR "ARV" OR "Fármacos Antirretrovirais" OR "Medicamentos Antirretrovirais" OR "Anti-Retroviral Agents" OR "Antirretrovirales"

#2 "Criança" OR "Crianças" OR "Child" OR "Niño" OR "Adolescente" OR "Adolescência" OR "Adolescentes" OR "Jovem" OR "Jovens" OR "Juventude" OR "Adolescent" OR "Adulto Jovem" OR "Young Adult" OR "Adulto Joven"

#3 "Crescimento " OR " Crescimento Corporal" OR " Growth" OR " Crecimiento" OR " Tamanho Corporal" OR " Body Size" OR " Tamaño Corporal" OR "Composição Corporal" OR "Body Composition" OR "Composición Corporal" OR "Distribuição da Gordura Corporal" OR "Body Fat Distribution" OR "Distribución de la Grasa Corporal" OR "Obesidade" OR "Obesity" OR "Obesidad" OR "Sobrepeso" OR "Overweight"

Results: 0

1.5 Web of Science

#1 human immunodeficiency virus OR human immunodeficiency virus infection OR human immunodeficiency virus 1 OR human immunodeficiency virus 2 OR antiretrovirus agent

#2 infant OR child OR adolescent OR young adult

#3 growth OR body size OR body composition OR body fat distribution OR obesity OR overweight

#1 AND #2 and #3

Results: 1.152
